# Cutaneous vasculitis in autoinflammatory diseases

**DOI:** 10.1111/1346-8138.17030

**Published:** 2023-11-13

**Authors:** Ko‐Ron Chen

**Affiliations:** ^1^ Meguro Chen Dermatology Clinic Tokyo Japan

**Keywords:** AIDs, cutaneous vasculitis, DADA2, FMF, livedo racemosa, VEXAS

## Abstract

Autoinflammatory diseases (AIDs) characterized by recurrent episodes of localized or systemic inflammation are disorders of the innate immune system. Skin lesions are commonly found in AIDs and cutaneous vasculitis can coexist with AIDs and even present as the most striking feature. This review aims to focus on the frequent cutaneous vasculitis association in three monogenic AIDs including familial Mediterranean fever (FMF), deficiency of adenosine deaminase type 2 (DADA2), and the recently identified adult‐onset VEXAS (vacuoles, E1 enzyme, X‐linked, autoinflammatory, somatic) syndrome. Cutaneous vasculitis in FMF is characterized by: (1) small‐vessel vasculitis similar to IgA vasculitis with palpable purpura but increased intussusception complication and less vascular IgA deposit, and (2) cutaneous arteritis–like vasculitis presenting as subcutaneous nodules most often with higher glomerular involvement. DADA2 has a wide spectrum of clinical presentations ranging from fatal systemic vasculitis with multiple strokes, especially in pediatric patients, to limited cutaneous disease in middle‐aged patients. DADA2 shares similar clinical and histopathological features with polyarteritis nodosa (PAN). As a result, DADA2 is commonly initially misdiagnosed as childhood PAN. Livedo racemosa reveals the most common cutaneous manifestation of cutaneous vasculitis in patients with DADA2. VEXAS syndrome is a life‐threatening disease. A diagnosis of VEXAS syndrome should be strongly considered or could be made in patients with skin lesions characterized by Sweet syndrome–like eruption, livedo racemosa, concomitant relapsing polychondritis, deep venous thrombosis, pulmonary involvement, and progressive hematologic abnormalities such as myelodysplastic syndrome with a unique finding of cytoplasmic vacuoles in myeloid and erythroid precursor cells from bone marrow aspirate smear. As skin involvement is common in AIDs and may present as the most frequent manifestation, especially in DADA2 (70% to 90%) and VEXAS syndrome (83% to 91%), dermatologists play a crucial role in contributing to the early diagnosis of these AIDs with early initiation of the appropriate therapy to avoid progressing fatal outcomes.

## 1 INTRODUCTION

Autoinflammatory diseases (AIDs), first described in 1999,^1^ are a group of rare diseases with recurrent episodes of uncontrolled systemic inflammation diseases of the innate immune system characterized by severe stimulation of inflammatory pathways without involvement of antigen‐directed autoimmunity with high‐titer autoantibodies and antigen‐specific T and B cells.^2^ Since then, over the past 20 years, numbers of both monogenic autoinflammatory and polygenic inflammatory disorders have been in continuous expansion^3^, ^4^ and notable developments in the field of autoinflammation and research in this area have led to an increased understanding of disease mechanisms and management of these conditions.^5^, ^6^

Monogenic AIDs with a specific genetic mutation represent loss‐of‐function mutations in genes that suppress inflammation or gain‐of‐function mutations in genes that propagate inflammation, resulting in immune activation spontaneously or with minimal triggering. It is important to note that monogenic autoinflammatory conditions often occur in childhood, but they can present in adulthood due to either de novo mutations or the presence of mosaicism.^7^, ^8^, ^9^ Multigenic autoinflammatory syndromes are not associated with a single identified genetic mutation and their genetic characterization is complex due to the involvement of multiple genes.^10^

Dermatologic involvement is common in monogenic AIDs and may represent the predominant and the initial event in some of them.^11^ The various cutaneous manifestations can be classified as: (1) maculopapular lesions or inflammatory plaques; (2) urticarial lesions; (3) pustular, pyogenic, or neutrophilic dermatosis‐like lesions; (4) panniculitis or subcutaneous nodules; (5) vasculitis or vasculopathy; (6) hyperkeratotic lesions; (7) hyperpigmented lesions; (8) bullous lesions; and (9) aphthous lesions. Coexistence of the different cutaneous manifestations is commonly found in a single patient on the same or different occasions.^11^

Cutaneous vasculitis can be a coexisting disease seen with AIDs and may also be one of the most striking features of certain types of AIDs. Cutaneous vasculitis has been identified in several monogenic AIDs^12^ such as familial Mediterranean fever (FMF), cryopyrin‐associated periodic fever syndrome, mevalonate kinase deficiency/hyperimmunoglobulin D syndrome, tumor necrosis factor receptor–associated periodic syndrome, pyogenic arthritis with pyoderma gangrenosum and acne syndrome/pyoderma gangrenosum with acne and hidradenitis suppurativa syndrome, haploinsufficiency of A20, deficiency of adenosine deaminase 2 (DADA2),^12^ and a recently identified adult‐onset VEXAS (vacuoles, E1 enzyme, X‐linked, autoinflammatory, somatic) syndrome^9^ presenting with predominant cutaneous manifestations.^13^ Cutaneous vasculitis in polygenic AID is most often found in Behçet disease.^12^

Although it is often difficult to make an early diagnosis of AIDs and collaboration with hematologists, rheumatologists, neurologists, and a team of genetic analysis is required, the frequent association of skin lesions encourages dermatologists to play an important role in providing early diagnostic clues by evaluating both the unique skin manifestations clinically in association with histopathological findings through skin biopsy.

This review will focus on the most common cutaneous vasculitis in three monogenic inflammatory diseases including FMF, DADA2, and VEXAS syndrome.

## 2 FAMILIAL MEDITERRANEAN FEVER

FMF, which mainly affecting people from Mediterranean countries, is one of the most prevalent monogenic AID. The majority of these pathogenic variants are located in exon 10 and are associated with mutations in the MEFV gene,^14^ MEFV encodes pyrin, a protein expressed in neutrophils and monocytes and regulates innate immunity through an inflammasome that leads to the production of interleukin (IL) 1β.^15^ FMF typically presents with recurrent episodes of fever, associated with acute abdominal pain and large joint arthritis that last 1 to 3 days and develops symptoms most often before the third decade of life.^16^ Flares recur periodically between once a week and once a year with symptom‐free intervals in between flares.^17^, ^18^, ^19^

Fever attacks occur suddenly with high‐grade fever (38.5–40°C) and severe asthenia.^20^ Moderate to severe abdominal pain occurs in 95% of the patients and is secondary to acute generalized peritonitis.^21^

The diagnosis of FMF has been based on clinical criteria that include frequent symptoms such as abdominal and thoracic pain, family history, and response to treatment with colchicine.^22^

A newer set of criteria requiring the presence of at least two of the following five criteria have been proposed: (1) fever >38°C, (2) abdominal pain, (3) chest pain, (4) arthritis, and (5) family history of FMF. This resulted in an improved specificity to 94% with a sensitivity of 86% for FMF diagnosis in a Turkish population based on a study that included 170 affected children and 141 healthy controls.^23^ Nevertheless, the finding of mutations in the MEFV gene is mandatory for a definitive diagnosis of FMF. During FMF flares, laboratory examinations typically indicate leukocytosis and increased acute‐phase reactants, such as erythrocyte sedimentation rate and C‐reactive protein.^16^

Erysipelas‐like erythema, the only pathognomonic cutaneous marker of FMF^11^, ^16^, ^24^ typically occurring on the lower extremities, with recurrent well‐demarcated erythematous plaque and spontaneously resolving within 2 to 3 days. It is characterized by mild papillary dermal edema, dilated vessels, and sparse perivascular mononuclear cell infiltrate admixed with neutrophils and nuclear dusts histopathologically,^24^ which may frequently be misdiagnosed as cellulitis.^16^

Numerous reports suggest a higher frequency of vasculitis in patients with FMF compared with the general population.^25^, ^26^, ^27^, ^28^, ^29^, ^30^ Cutaneous vasculitis in FMF, presenting as: (1) FMF‐associated IgA vasculitis, a dermal small‐vessel vasculitis; and (2) FMF‐associated polyarteritis nodosa (PAN), a dermosubcutaneous to subcutaneous medium‐sized arteritis, are the most frequent forms of vasculitis in FMF.^25^, ^26^, ^27^, ^29^, ^30^ A recent French study of FMF vasculitis in 22 patients also revealed that both PAN (10 of 22 [46%]) and IgA vasculitis (8 of 22 [36%]) are the most frequent forms of vasculitis in FMF, with a higher frequency for FMF‐associated PAN than FMF‐associated IgA vasculitis.^30^

## 3 FMF‐ASSOCIATED IGA VASCULITIS

The prevalence of IgA vasculitis in FMF ranging from 2.7% to 7% was significantly higher than that of idiopathic IgA vasculitis without FMF which is ranging from 0.05% to 0.8% in controls from the same health centers.^26^, ^27^ The median age at diagnosis of FMF‐associated IgA vasculitis has been shown to be 10 years^29^ to 13.5 years,^30^ while the median age at diagnosis of FMF was 6.8 years^29^ to 9 years^30^ (4.5 to 15 years), about 4 years proceeded the onset of IgA vasculitis.^29^, ^30^ Clinical manifestations similar to those in idiopathic IgA vasculitis without FMA showed purpura (100%)^25^, ^26^, ^27^, ^28^, ^29^, ^30^, ^31^, ^32^ on the legs in all patients, followed by renal involvement (52% to 80%), abdominal pain (50% to 71.7%), and arthralgia/arthritis (33% to 71.7%); however, these patients revealed a higher complication of intussusception (8.7%) and possibly less IgA vascular deposit than patients with idiopathic IgA vasculitis.^29^, ^30^

## 4 FMF‐ASSOCIATED PAN

A striking difference in the mean onset age of 18 years in patients with FMF‐associated PAN and that of 50 years in patients with idiopathic PAN without FMF has been shown,^27^, ^29^ but they were older than those with FMF‐associated IgA vasculitis (mean age, 10.5 years).^29^ Patients with FMF‐associated PAN showed a significantly higher prevalence of 1.3% than patients with PAN without FMF (0.004%).^29^

Cutaneous manifestations appeared as subcutaneous nodules and infiltrated erythema similar to those in cutaneous arteritis, followed by livedo and purpura, and lesions of erysipelas‐like erythema without histological evidence of vasculitis could be found. Biopsy should be taken from the subcutaneous nodules or infiltrated erythema to obtain the definitive histopathological evidence of arteritis.

Renal involvement (49%) in FMF‐associated PAN characterized by suspected glomerular involvement (34%), perirenal hematoma (49%), and higher central nervous system involvement (31%) present as the striking differences with idiopathic PAN sparing glomerular and perirenal involvement.^29^, ^30^ Systemic corticosteroids were the most often used agents for treating vasculitis in patients with FMF.

## 5 DEFICIENCY OF ADENOSINE DEAMINASE 2

DADA2 was initially described in 2014 as a syndrome of small‐ and medium‐vessel vasculitis/vasculopathy manifesting as recurrent episodes of fever, early‐onset lacunar strokes, and cutaneous involvement including livedo racemosa, Raynaud phenomenon, and PAN. DADA2 is caused by biallelic loss of function mutations in the ADA2 gene (formerly CECR1).^33^, ^34^

The extracellular enzyme adenosine deaminase 2 (ADA2) that converts adenosine and 2′‐deoxyadenosine to inosine and 2′‐deoxyinosine, respectively, is encoded by the ADA2 gene and is mainly expressed by monocytes/cells of the myeloid lineage.^35^ It promotes the proliferation of monocytes and induces the differentiation of macrophages. Patients with a DADA2 have defective differentiation of M2 macrophages (anti‐inflammatory effects).^36^ Consequently, DADA2 leads to increased M1 macrophages that release proinflammatory cytokines from monocytes and macrophages. These cytokines induce inflammation, damage the endothelial cells, and injure the vessel walls.^35^, ^36^, ^37^ DADA2 also regulates the activation of neutrophils as neutrophils express adenosine receptors, and decreased ADA2 activity may cause endothelial damage by increased activation of neutrophils chronically.^37^

The onset of most monogenic AIDs mainly appear in childhood; however, clinical signs and symptoms of DADA2 syndrome may also start in adulthood and up to 8.5% of patients may have the first signs or symptoms after 18 years old.^38^

DADA2 has a wide spectrum of clinical presentations that range from fatal systemic vasculitis with multiple strokes, especially in pediatric patients, to limited cutaneous disease in middle‐aged patients.^39^

Clinical manifestations of DADA2 can be grouped into three categories: (1) inflammatory (vasculitis/vasculopathy/rash); (2) immune dysregulation; and (3) hematologic abnormalities. Although patients often present with primary features of one of these categories, considerable overlapping of the subgroups has been observed.

Skin involvement is the most common clinical manifestation, ranging from 90%^40^ (52 to 58) to 67.9%^38^ (257 to 378) of patients with DADA2. Among them, livedo racemosa is the most common skin manifestation (43 of 58 [74%]^40^ and 180 of 257 [70%]),^38^ followed by cutaneous nodules resembling PAN/nodules (33 of 58 [57%])^40^ maculopapular erythema, livedoid lesions, Raynaud phenomena, and digital cyanosis with gangrene ulcers were occasionally found.^40^

Histopathology of the skin lesions ranges from nonvasculitic characterized by thrombovasculopathy to vasculitic characterized by medium‐sized arteritis resembling PAN with mixed features of mononuclear cells, neutrophils, and nuclear dusts in the infiltrate and destruction and fibrinoid necrosis of the affected vessel wall.^40^ Dermal to subcutaneous small‐vessel vasculitis may also be observed.^40^

DADA2 shares many similar clinical and histopathological features with PAN, and, as a result, DADA2 may be initially misdiagnosed as childhood PAN.^39^, ^41^, ^42^ It has been reported that about 25% of patients with DADA2 have been misdiagnosed as having childhood PAN.^41^

Huang et al.^41^ reported the following features that may help to distinguish DADA2 from PAN: (1) DADA2 usually presents earlier and at younger ages; (2) skin manifestations are more commonly seen in childhood PAN and DADA2 compared with adult PAN; (3) the livedo presentation of the skin is more common in DADA2 than PAN; (4) the peripheral nervous system is more involved in adult PAN; (5) central nervous system involvement such as ischemic stroke and brain bleeding occur more often in DADA2; and (6) patients with PAN usually have increased white blood cell and platelet counts, while patients with DADA2 patients usually have decreased immunoglobulin (IgA, IgM, and IgG) levels and decreased platelet and white blood cell counts.^41^

Screening for ADA2 mutations has been recommended in patients with signs and symptoms of vasculopathy and vasculitis (similar to PAN), particularly in cases with early‐onset clinical features or a history of strokes, inflammatory diseases, and family history.^36^, ^43^ Early diagnosis and therapy with tumor necrosis factor α inhibitors are important in preventing severe complications as some of these complications can be prevented.^36^

Enzymatic testing in addition to genetic testing is the standard clinical testing for diagnosing DADA2.^44^ DADA2 enzymatic testing of <5% of normal or undetectable ADA2 activity confirms the diagnosis of DADA2.^44^

In addition, measuring serum ADA2 activity before genetic testing is more cost‐beneficial.^41^, ^45^

The majority of patients with DADA2 present before the age of 10 years and approximately 25% of patients present before the age of 1 year. About 8% of patients with DADA2 die at a young age (<30 years). The majority of the patients die due to complications caused by recurrent stroke or infection.^46^, ^47^

The early diagnosis and treatment of DADA2 are crucial as the clinical features could be potentially life‐threatening but treatable. Antitumor necrosis factor, the first‐line treatment for most cases of vasculitis, has shown proven potency in decreasing rates of stroke occurrence and levels of inflammatory markers.^36^

### 5.1 VEXAS syndrome

VEXAS syndrome is a recently described adult‐onset AID occurring almost exclusively in men at a mean onset age of 64 to 71 years,^9^, ^13^, ^48^, ^49^ although it was recently found in scattered cases of female patients.^50^ VEXAS syndrome is a severe and life‐threatening disease with high mortality rates and 5‐year survival of 63%^13^ to 50%.^49^ VEXAS syndrome results from a somatic mutation affecting UBA1, a gene that codes for the E1 ubiquitin‐activating protein. Loss of UBA1 leads to a broad range of inflammatory conditions, and the clinical course is often refractive to therapy.^9^

Skin lesions are the most common clinical manifestations, occurring in 84% to 92% of cases,^9^, ^13^, ^48^, ^51^ followed by noninfectious fever (65% to 92%),^9^, ^13^, ^48^ lung involvement (50% to 90%),^9^, ^13^, ^48^, ^51^ hematological diseases (50% to 90%),^9^, ^13^, ^48^ weight loss (55% to 76%),^13^, ^48^ ocular symptoms (38.8% to 64%)^13^, ^48^, ^52^ relapsing polychondritis (36.4% to 64%),^9^, ^13^, ^48^ deep venous thrombosis (34.7% to 56%),^48^, ^53^, ^54^ arthralgias (28% to 52%),^13^, ^48^ and lymphadenopathy (32% to 35%).^13^, ^48^

## 6 KEY CLINICAL MANIFESTATIONS OF VEXAS SYNDROME

### 6.1 Relapsing polychondritis

**FIGURE 1:**
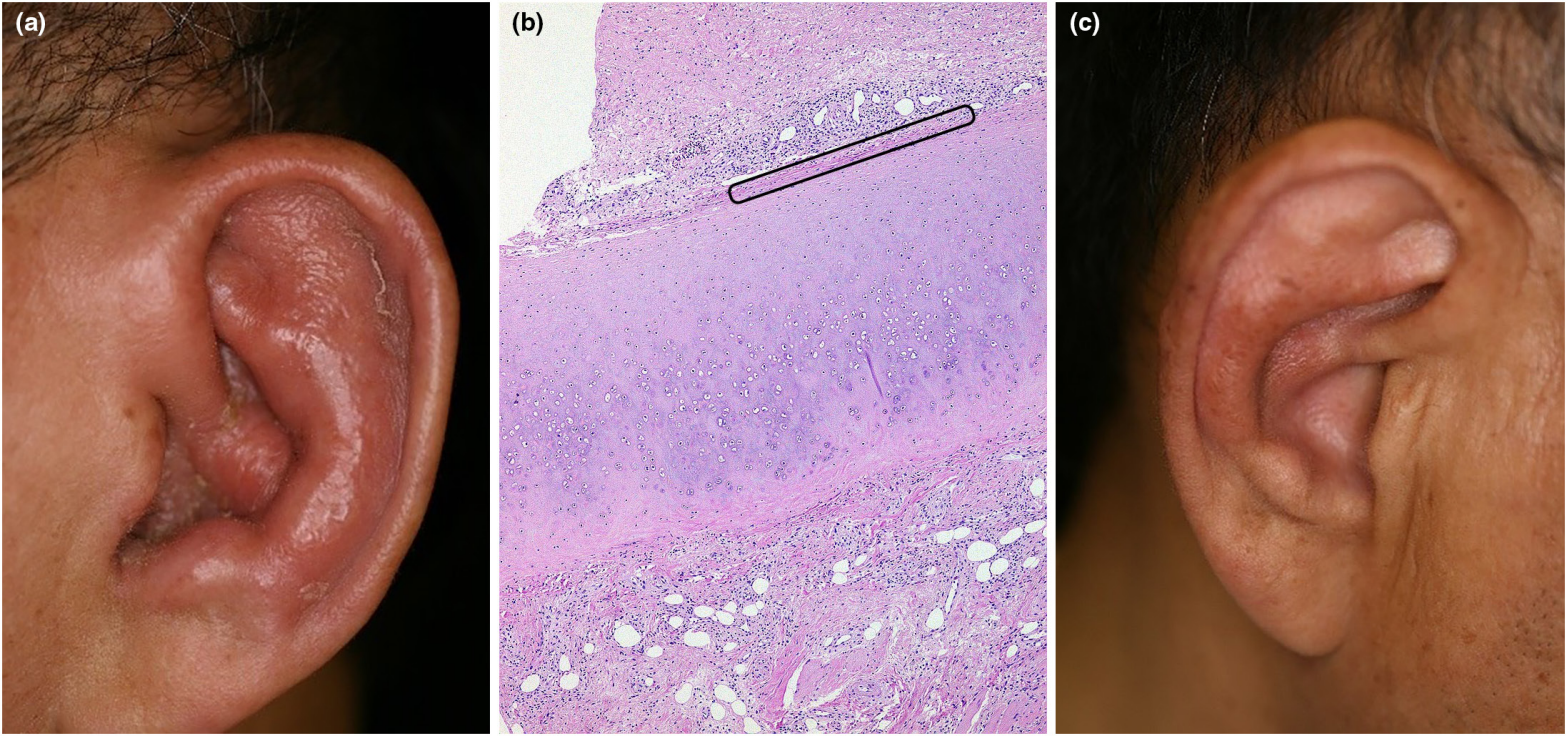
(a) A 65‐year‐old male was first seen with a chief complaint of painful swelling on his left ear. (b) Biopsy from the left ear revealed marked infiltrates of neutrophils with debris in and around the affected cartilage (rectangle) consistent with the histopathologic features of chondritis (magnification, ×40). (c) Early development of polychondritis of another ear was seen 3 months after his first visit.

Up to 64% of patients reveal relapsing polychondritis most often involving cartilage of the ears (Figure 1) and nose (36%,^13^ 44%,^48^ and 64%^9^); even the respiratory trachea may be involved. VEXAS syndrome should be considered in patients with unexplained relapsing polychondritis and concomitant macrocytic anemia characterized by peripheral cytopenia and increased mean corpuscular volume.^55^

### 6.2 Skin manifestations

**FIGURE 2:**
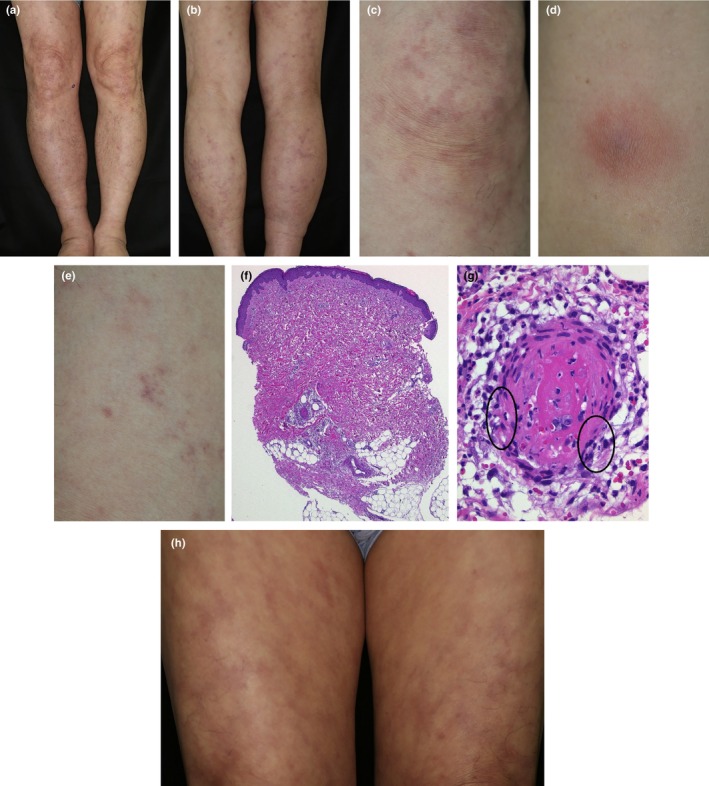
Various cutaneous manifestations on the lower extremities were found 15 months after his first visit. (a, b) Livedo racemosa on both legs. (c) Livedo racemosa mixed with infiltrated erythema at higher magnification. (d) Tender swollen erythema appeared on the same lesional leg. (e) Erythematous purpuric lesions could be identified on the same lesional leg. (f) Biopsy from livedo racemosa revealed arteritis at dermosubcutaneous junction (magnification, ×40). (g) Higher magnification showed infiltration of neutrophils in and around the disrupted arterial wall (circles) with luminal fibrin and marked extravasation of erythrocytes (magnification, ×400). (h) Marked development of livedo racemosa mixed with residual tender infiltrated erythema on both thighs was seen 3 months after onset of skin lesions of a–e.

Skin manifestations are the most frequent clinical expression of VEXAS syndrome (97 of 116 [84%],^13^ 22 of 25 [88%],^48^ 41 to 45 [91%],^51^ and 23 to 25 [92%]^9^) with various cutaneous manifestations occurring on different occasions, including livedo racemosa (Figure 2a–c,h), infiltrated erythema (Figure 2c,h), tender swollen erythema (Figure 2d), erythematous purpuric lesions (Figure 2e), neutrophilic urticarial dermatosis, urticaria, erythema nodosum, erythematous plaques, subcutaneous reddish purple tender nodules, and Sweet syndrome–like eruptions with tender red or violaceous edematous papules on the neck and trunk (Figure 4). The most commonly reported cutaneous manifestation of VEXAS syndrome is neutrophilic dermatosis.^13^, ^48^ Livedo racemosa with histopathologically proven arteritis (Figure 2f,g) is also a commonly concomitant skin manifestation. A study of eight VEXAS cases revealed Sweet syndrome–like dermatosis in all, myelodysplastic syndrome in six, and livedo racemosa on the legs in three of the eight cases.^56^

Our 65‐year‐old male patient with VEXAS initially developed relapsing polychondritis (Figure 1) followed by the development of various skin lesions on different occasions, including livedo racemosa (Figure 2a–c,h), infiltrated erythema (Figure 2c,h), tender swollen erythema (Figure 2d) and erythematous purpuric lesions (Figure 2e) 1 year later, and appearance of Sweet syndrome–like dermatosis (Figure 4) 23 months after his first visit.

Skin involvement may appear before or at the time of other clinical features of VEXAS syndrome.^48^, ^56^

### 6.3 Vasculitis

Vasculitis (30%) cases included the following:

- Large‐vessel vasculitis: giant cell arteritis (4%).^9^
- Medium‐sized vessel vasculitis (5%^48^ to 12%^9^): medium‐sized vessel vasculitis in skin is commonly identified as arteritis at dermosubcutaneous junction (Figure 2f,g) and subcutaneous arteritis less often.
- Small‐vessel vasculitis (20%^48^, ^57^): dermal to subcutaneous leukocytoclastic vasculitis is most commonly found.^48^, ^57^, ^58^, ^59^ Cases with coexistent IgA vasculitis^60^ and antineutrophil cytoplasmic antibodies–associated small‐vessel vasculitis^61^ have also been reported.
- Deep dermal to subcutaneous thrombophlebitis^48^, ^56^ (4 of 25 [16%]^48^).
- Cutaneous vasculitis of dermal to subcutaneous small‐vessel vasculitis (leukocytoclastic vasculitis) most often presents as erythematous purpuric lesions (Figure 2e) or erythematous papules, while arteritis at the dermosubcutaneous junction (Figure 2f) or subcutis in VEXAS syndrome usually presents as livedo racemosa (Figure 2a–c,g) or subcutaneous reddish purple tender nodules.^57^

### 6.4 Ocular involvement

**FIGURE 3:**
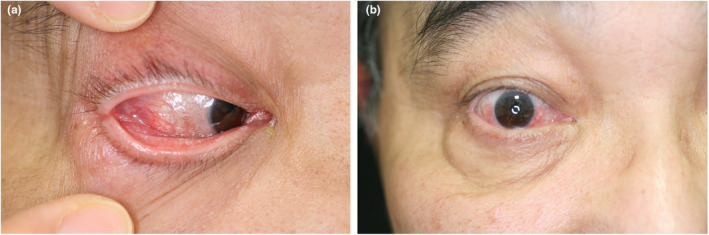
(a) The patient developed episcleritis 18 months after his first visit. (b) Moderate improvement of the ocular inflammation was achieved after 2 months of glucocorticoid treatment.

Ocular inflammation with periocular erythema and edema occurs in up to 64% of patients (41%^13^, ^52^ to 64%^48^) and most often reveals episcleritis (12.1%) (Figure 3) followed by uveitis (9.5%), scleritis (8.6%), orbital mass (3.4%), and blepharitis.^52^

### 6.5 Pulmonary involvement

Pulmonary involvement is very common (50% to 90%),^9^, ^13^, ^51^ with more than half of patients characterized by pulmonary infiltrates, pleural effusion, and multifocal ground‐glass opacities (Figure 5), accounting for the most frequent disease manifestations. Pulmonary fibrosis, bronchiolitis obliterans, and neutrophilic alveolitis have also been described in patients with VEXAS.^9^, ^13^, ^51^ Parenchymal opacities (Figure 5) are seen on chest computed tomography in three‐fourths of the patients.^51^ Most patients respond to glucocorticoid therapy, but relapses occur at lower doses.^51^

### 6.6 Hematological disorders

Hematological disorders (36%,^48^ 44%,^9^ 60%,^13^ and 75%^56^) commonly presenting as macrocytic anemia (96%^9^, ^48^) or myelodysplastic syndrome (MDS) reveal a key clinical manifestation of VEXAS syndrome. MDS has been diagnosed at a high frequency in patients with VEXAS syndrome (6 of 25 [25%],^9^ 3 of 10 [30%],^62^ 6 of 11 [55%],^63^ and 6 of 8 [75%]^56^).

The significant findings of cytoplasmic vacuoles in myeloid and erythroid precursor cells from bone marrow aspirate smear (Figure 6) suggest a diagnostic clue for VEXAS syndrome.

### 6.7 Deep venous thrombosis or pulmonary embolism

Venous thrombosis (35%,^13^ 36.4%,^54^ 56%,^53^ 44%,^9^ and 52%^48^), markedly higher than arterial thrombosis (1.6%), develops in up to 56%^8^ of patients and most commonly occurrs in deep veins of the lower extremities, which may even extend intensively to the inferior vena cava, as was found in our case. In contrast, pulmonary embolism is relatively more rare than deep venous thrombosis.^53^, ^54^

## 7 DIAGNOSIS

**FIGURE 4:**
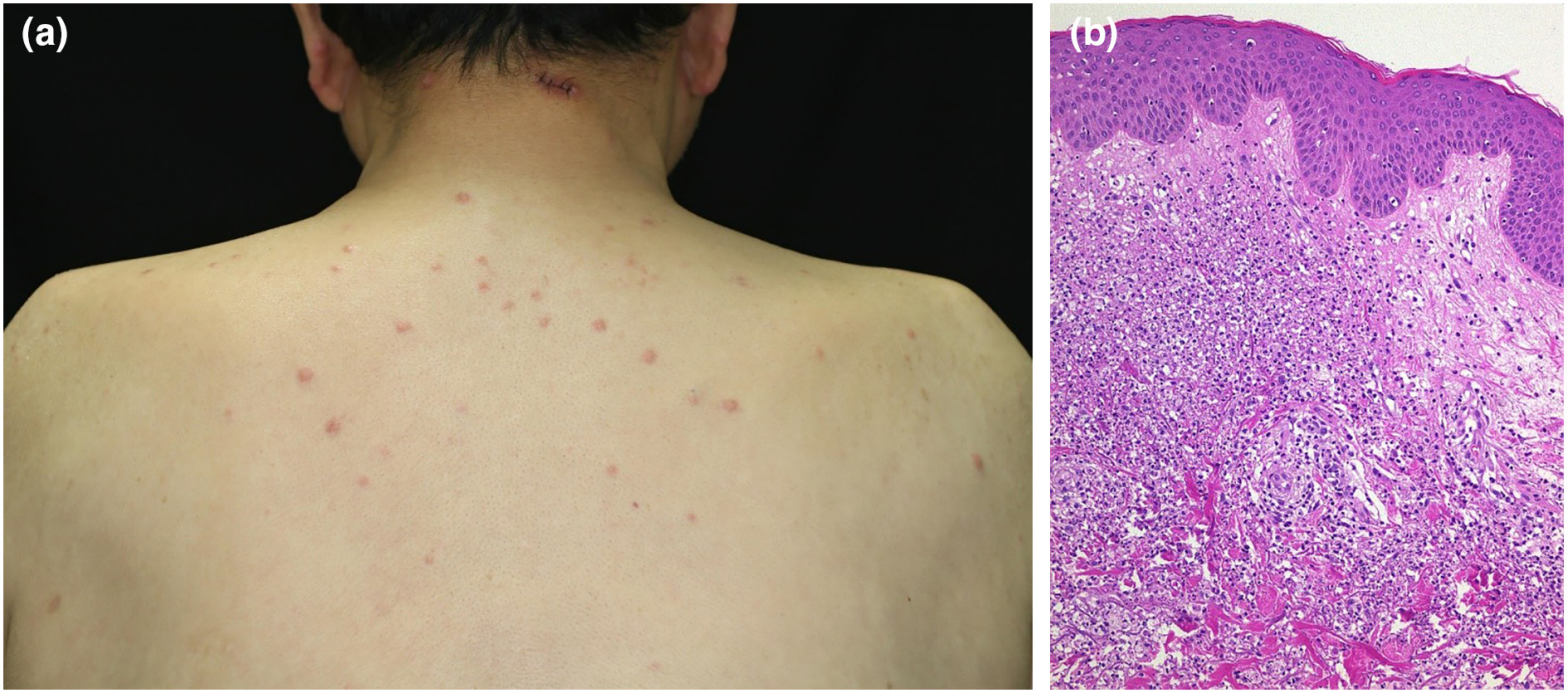
(a) Sweet syndrome–like swollen erythematous papules developed on the upper back and neck 23 months after the patient's first visit. (b) Skin biopsy showed marked infiltration of neutrophils mixed with nuclear debris at the edematous papillary dermis and mid dermis consistent with the histopathology of Sweet syndrome (magnification, ×100).

**FIGURE 5:**
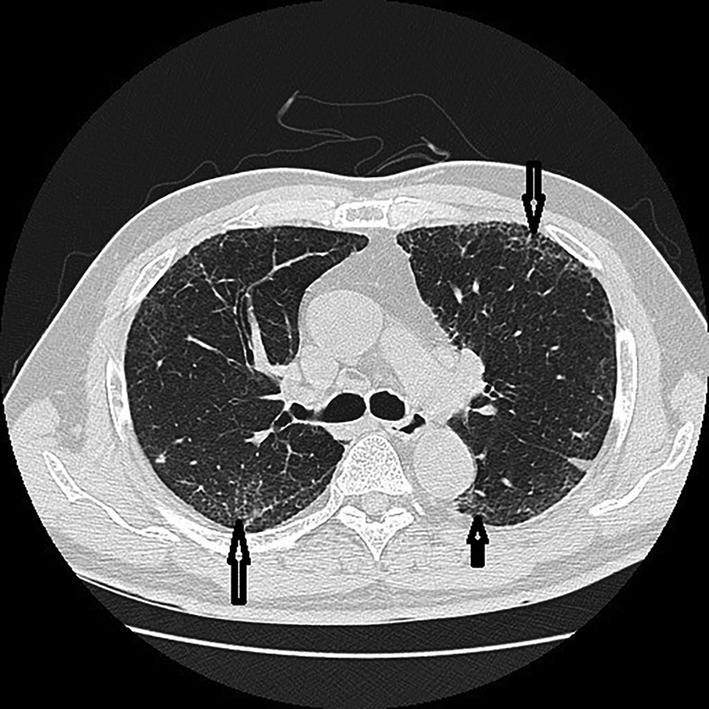
The patient also developed interstitial pneumonia with multifocal ground‐glass opacities (arrows) 2 years after his first visit.

**FIGURE 6:**
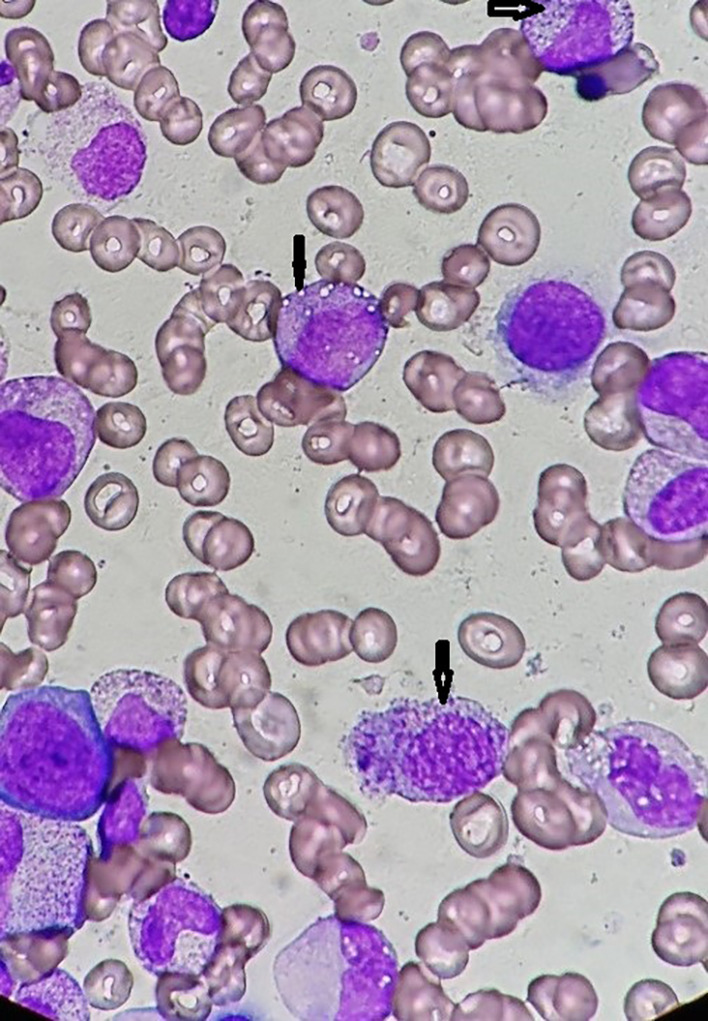
Three years after the patient's first visit, he developed myelodysplastic syndrome and a bone marrow aspirate smear showed marked cytoplasmic vacuoles (arrows) in myeloid and erythroid precursor cells (May‐Giemsa stain) (magnification, ×1000).

The definite diagnosis of this syndrome depends solely on the presence of UBA1 mutations confirmed by the Sanger sequencing method.^64^ Nevertheless, a diagnosis of VEXAS syndrome should be strongly considered and could be made based on their specific clinical manifestations, especially in patients who followed a treatment‐refractory course and revealed symptoms of relapsing polychondritis (Figure 1), various unique skin lesions such as livedo racemosa on the lower extremities (Figure 2), and Sweet syndrome–like eruption on the neck and trunk (Figure 4) on different occasions with concomitant ocular inflammation (Figure 3), pulmonary involvement (Figure 5), and progressive hematologic abnormalities such as macrocytic anemia or MDS^9^, ^13^, ^48^, ^56^ (Figure 6).

Diagnostic criteria for VEXAS syndrome based on score‐guided clinical assessments should be established in the future, as genetic sequencing analysis has its limitations and is only available in certain institutions.

The clinical manifestations listed above as the key specific manifestations for VEXAS syndrome, in addition to the unique finding of vacuoles in myeloid and erythroid progenitors from bone marrow biopsy or aspirate smear (Figure 6), strongly suggest the diagnosis of VEXS syndrome and could lead to the early diagnosis and treatment for managing this progressing and fatal condition earlier.

## 8 TREATMENT

To date, various treatments, including: (1) glucocorticoids, conventional disease‐modifying antirheumatic drugs^64^ (methotrexate, mycophenolate mofetil, azathioprine); (2) biotechnological agents targeting IL‐1 and IL‐6 and Janus kinase inhibitors^65^, ^66^; (3) allogeneic hematopoietic stem cell transplantation^49^, ^67^; or (4) targeting the etiologic UBA1 clone or inhibiting the inflammatory cascade,^68^ have been reported to achieve certain acceptable results. Nevertheless, there still remains no consensus on an optimal treatment strategy, and a prospective evaluation of treatment efficacy is needed to define optimal clinical management.

## 9 CONCLUSION

As skin involvement is common in AIDs and may present as the most frequent and initial manifestation in both DADA2 (70% to 90%) and VEXAS syndrome (83% to 91%), dermatologists play a crucial role in the early diagnosis of both DADA2 and VEXAS syndrome and may contribute to the early management of these rare but progressing and fatal AIDs.

## CONFLICT OF INTEREST STATEMENT

None declared.
